# Transcripts from the *src-1(cj293)* mutant can encode a SRC-1 molecule lacking the SH2 domain in *Caenorhabditis elegans*

**DOI:** 10.17912/micropub.biology.001537

**Published:** 2025-03-12

**Authors:** Snehal S Mahadik, Erik A. Lundquist

**Affiliations:** 1 Molecular Biosciences, University of Kansas, Lawrence, Kansas, United States

## Abstract

Previous studies suggest that the *src-1 ( cj293 )* mutation is an activated *src-1* allele in *C. elegans* with the potential to encode a molecule lacking the SH2 domain. *src-1 ( cj293 )* is a deletion with breakpoints in introns 3 and 5, deleting exons 4 and 5, which encode the SH2 domain. If exon 3 is spliced to exon 6, the reading frame is maintained. Here, RNA seq of *src-1 ( cj293 )* mutants showed that the exon 3 to exon 6 splice does not occur in *src-1 (+)* but is robustly present in *src-1 ( cj293 )* . Thus, *src-1 ( cj293 )* produces a transcript that can encode a SRC-1 molecular lacking the SH2 domain, which leads to overactive SRC-1 in growth cones of VD neurons during their outgrowth ( *i.e.* *src-1 ( cj293 )* might be a constitutively-active mutation).

**Figure 1. Transcripts from the src-1 locus in src-1(+) and src-1(cj293)/+:**
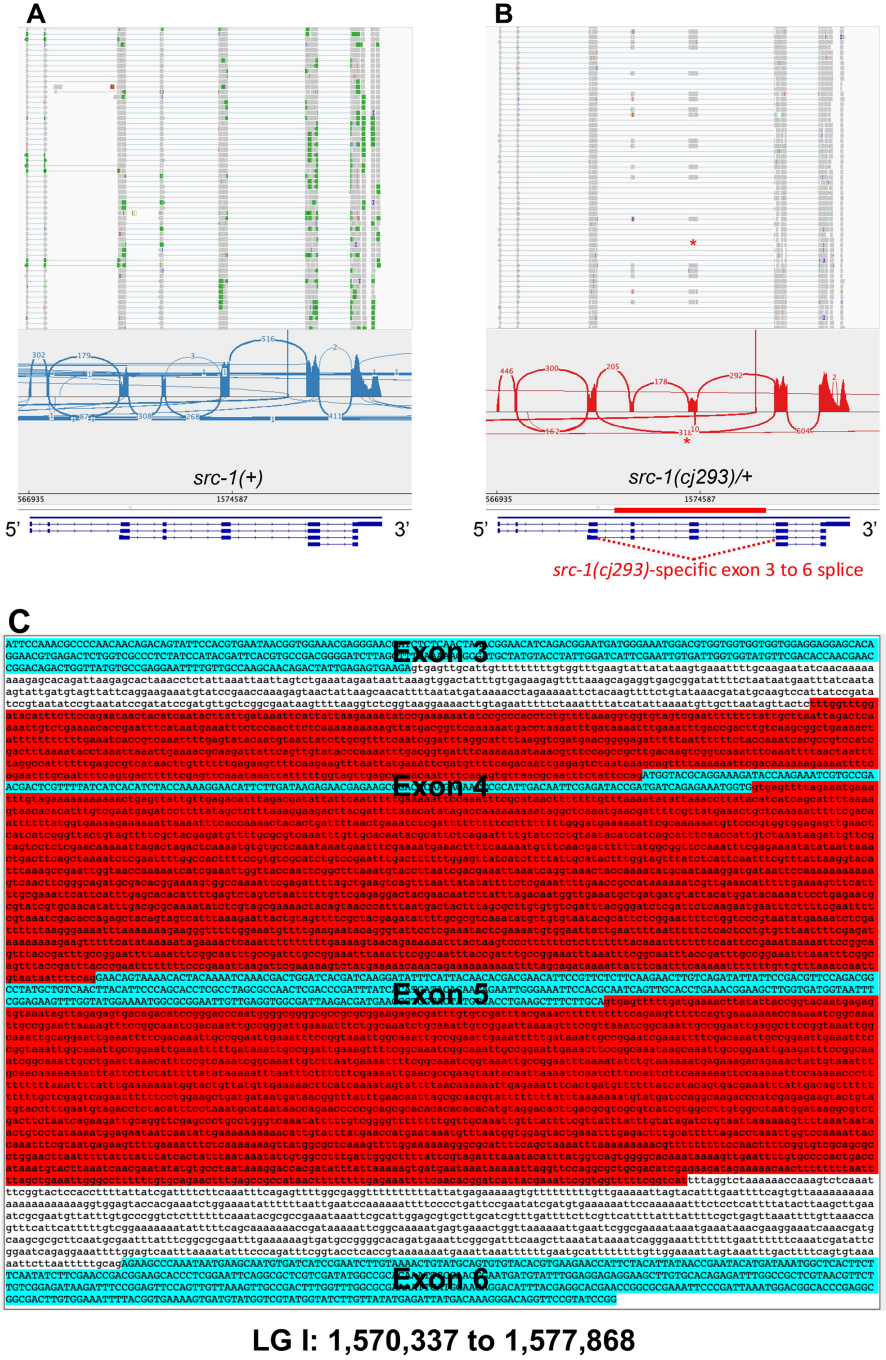
Alignments of RNA seq to the *src-1* locus in A) *src-1(+)* and B) *src-1(cj293)* . At the top are representative alignments from the Integrated Genome Viewer. The asterisk in *src-1(cj293)/+* indicates a group of exon 3 to exon 6 splices, which did not occur in *src-1(+)* . Shown below the alignments are Sashimi plots of splicing at the *src-1* locus, generated in the Integrated Genome Viewer. The 318 exon 3 to exon exon 6 splices are indicated with an asterisk. The structure of the *src-1* locus is shown at the bottom. The extent of the *src-1(cj293)* deletion is indicated with a red bar. 5' to 3' orientation of the locus is indicated. The *src-1(cj293)-* specific exon 3 to 6 splice is indicated with a dashed red line below the gene model. C) The sequence of the *src-1* locus from exon 3 to exon 6 (bases 1,570,337 to 1,577,868 on LGI). The region deleted in *src-1(cj293)* is red, and the exons are cyan. Exons 4 and 5 are removed by the *src-1(cj293)* deletion.

## Description

The SRC-1 /Src tyrosine kinase in *Caenorhabditis elegans* is required for embryonic development, cell migration, and axon guidance (Bei *et al.* 2002; Itoh *et al.* 2005; Lee *et al.* 2005; Sugioka and Sawa 2010; Masuda *et al.* 2012; Zhu *et al.* 2020; Mahadik *et al.* 2024). In developing VD axon growth cones, SRC-1 acts with the UNC-6 /Netrin receptor UNC-5 to inhibit growth cone protrusion (Mahadik *et al.* 2024). A precise deletion mutant of *src-1* had VD growth cones that displayed excessive protrusion resulting in axon guidance defects, similar to *unc-5* loss-of-function (Mahadik *et al.* 2024). The *src-1 ( cj293 )* in-frame deletion removes exons that encode the SH2 domain and an N-terminal portion of the kinase domain (Mahadik *et al.* 2024). *src-1 ( cj293 )* is predicted to encode a molecule lacking the SH2 domain and part of the kinase domain (Mahadik *et al.* 2024). Similar to the *src-1 (lq185)* precise deletion allele, *src-1 ( cj293 )* mutants display defects in embryonic development (Bei *et al.* 2002; Mahadik *et al.* 2024). However, *src-1 ( cj293 )* mutants displayed VD growth cones with reduced protrusion compared to *wild-type* , similar to *src-1 (+)* overexpression (Mahadik *et al.* 2024). This suggests that *src-1 ( cj293 )* might encode a constitutively-active SRC-1 molecule.

The breakpoints of the *src-1 ( cj293 )* mutation are in introns 3 and 5, removing exons 4 and 5 ( Figure 1A and B). If exon 3 is spliced to exon 6 (3-6), the reading frame is maintained, resulting in coding potential for a molecule lacking the SH2 domain and part of the kinase domain (Mahadik *et al.* 2024). RNA seq was conducted in heterozygous *src-1 ( cj293 )/+* animals. Reads were aligned to the *C. elegans* genome, and Sashimi plots were generated to illustrate splicing events. In animals with a *wild-type src-1 (+)* gene, the 3-6 splice did not occur ( Figure 1A ). However, in *src-1 ( cj293 )/+* animals, the 3-6 splice was common (318 times) ( Figure 1B ). The 3-6 splice products produced in *src-1 ( cj293 )* have the potential to produce a SRC-1 molecule missing the SH2 domain and part of the kinase domain. The catalytic residue of the kinase is not in the deleted region. Kinase function is likely active in *src-1 ( cj293 )* , as mutation of the catalytic residue (D381A) in *src-1 (syb7248)* resulted in a dominant phenotype resembling *src-1* precise deletion, with excessively-protrusive growth cones VD growth cones (Mahadik *et al.* 2024).

Autoinhibition of Src kinase activity is mediated by the SH2 domain, which binds to phosphorylated tyrosine 527, resulting in a closed, inactive conformation (reviewed in (Wagner *et al.* 2013)). This tyrosine is conserved in *C. elegans* SRC-1 (tyrosine 531), thus it might also be subject to autoinhibition by the SH2 domain. A SRC-1 molecule missing the SH2 domain may result in overactive kinase activity, consistent with the *src-1 ( cj293 )* overactive phenotype in the growth cone. It is also possible that *src-1 ( cj293 )* overactivity is due to other interactions that require the SH2 domain and/or the N-terminal portion of the kinase domain. *src-1 ( cj293 )* was not dominant for axon guidance defects, which would be expected of an activated molecule. Possibly, one copy of the activated allele is not sufficient to produce a phenotype in heterozygotes with *src-1 (+)* . In any case, these results are consistent with *src-1 ( cj293 )* producing an activated SRC-1 molecule lacking the SH2 domain and N-terminal portion of the kinase domain, which phenotypically, results in SRC-1 overactivity.

## Methods

RNA was isolated from mixed-stage animals as previously described (Tamayo et al. 2013; Paolillo et al. 2024). Poly-A selection and RNA seq library construction was conducted using the NEBnext stranded RNA seq kit. RNA seq libraries were made using the NEBNext stranded mRNA library kit. Sequencing was conducted on a Nextseq 2000 instrument with 150-bp paired-end sequencing. FASTQ files were processed using fastp (0.23.2) (Chen et al. 2018). Reads were aligned to the *C. elegans* reference genome [release WBcel235, version WBPS14 (WS271)] using HISAT2 (version 2.2.1) (Kim et al. 2015). BAM files from HISAT2 alignment were analyzed in the Integrated Genome Viewer (Robinson et al. 2011; Thorvaldsdottir et al. 2012), including Sashimi plots (Katz et al *.* 2010; Katz et al. 2015). Wormbase was used for *C. elegans* informatics (Sternberg et al., 2024)

## Reagents

Raw FASTQ reads for *src-1 (+)* and *src-1 ( cj293 )* were deposited in the Sequence Read Archive ( PRJNA1093133 and PRJNA1219192 , respectively). The *src-1 (+)* strain was LE5443 ( *unc-6 (lq154) X; juIs76 II* ). The balanced *src-1 ( cj293 )/+* strain was HR1275 ( *src-1 ( cj293 ) dpy-5 ( e61 )/ hT2 I; +/ hT2 III* ).
